# Framingham risk score in impaired glucose tolerant population: A sub analysis of Diabetes Prevention and Awareness Program of Pakistan

**DOI:** 10.12669/pjms.325.10448

**Published:** 2016

**Authors:** Asher Fawwad, Hassan Moin, Iftikhar Ahmed Siddiqui, Muhammad Zafar Iqbal Hydrie, Abdul Basit

**Affiliations:** 1 Asher Fawwad, PhD. Associate Professor, Baqai Medical University, Senior Research Scientist, Research Department, Baqai Institute of Diabetology and Endocrinology, Baqai Medical University, Karachi, Pakistan; 2 Hassan Moin, M.Sc. Statistician, Research Department, Baqai Institute of Diabetology and Endocrinology, Baqai Medical University, Karachi, Pakistan; 3 Iftikhar Ahmed Siddiqui, PhD. Chairman & Professor of Biochemistry, Department of Biochemistry, Baqai Institute of Diabetology and Endocrinology, Baqai Medical University, Karachi, Pakistan; 4 Muhammad Zafar Iqbal Hydrie, PhD, Postdoc. Assistant Professor, Department of Biochemistry, Hamdard College of Medicine and Dentistry, Hamdard University, Karachi, Pakistan; 5 Abdul Basit, FRCP. Professor of Medicine, Department of Medicine, Baqai Institute of Diabetology and Endocrinology, Baqai Medical University, Karachi, Pakistan

**Keywords:** Coronary artery disease, Framingham risk score, Impaired glucose tolerance, OGTT

## Abstract

**Objective:**

To assess the 10-year risk of coronary artery disease (CAD) in subjects with impaired glucose tolerance (IGT) using Framingham risk score.

**Methods:**

Data for this study was collected from Diabetes Prevention and Awareness Program. Primary prevention team visited different primary health care centers, factories, service organizations and offices within Karachi, Pakistan. IGT was diagnosed according to World Health Organization criteria after taking informed consent. Information regarding social-demography, dietary habits and physical activities were obtained by a designed questionnaire on one-to-one based interview. Framingham risk score (FRS) was used to assess risk of developing CAD.

**Results:**

A total of 315 subjects with IGT were recruited for the study. Mean age of subjects was 44.1 ± 9.8 years and mean BMI was 27.3 ± 5.0 kg/m^2^. Overall, 31.4% of the participants were at risk of having CAD. Males were 6.4 times and hypertensive subjects were 2.44 times more likely to have CAD in next 10 years.

**Conclusion:**

According to the findings of the study, male and hypertensive IGT subjects were more likely to develop CAD in next 10 years. Community based awareness programs are needed to educate people regarding healthy lifestyle in order to reduce the risk of IGT and CAD.

## INTRODUCTION

Coronary artery disease (CAD) is one of the leading causes of high mortality and morbidity in the world. Around 8.14 and 7.4 million deaths were recorded due to CAD in 2013 and 2012.^1^ Globally, CAD deaths represent about 30% of all deaths out of which 75% happened in the lower and middle-income countries.^2^

A study conducted in Pakistan population found a prevalence of about 6% and 4% in men and women respectively. It was also found in the study that one in five adults (aged ≥ 40 years) in urban parts of Pakistan may have CAD.^3^

In 1948, Framingham Heart Study, an ongoing study, was initiated with 5209 healthy adult subjects, aged 30 to 62 from the town of Framingham, Massachusetts.^4^ Prior to this study, very few facts were known about CAD.^5^ Most of the risk factors for CAD have been reported in this study. The most well-known factors are hypertension, smoking and diabetes mellitus.^6^ Other risk factors include older age, elevated systolic blood pressure, lack of exercise, obesity, male gender and dyslipidemia.^7^ Proper physical activities, weight management, appropriate diet and nutrition can play a vital role in prevention of CAD.^8^,^9^

Prevention strategies to modify risk factors can be implemented with the knowledge of risk factors to decrease morbidity and mortality. Majority of the cases with CAD can be prevented through modifiable risk factors.^10^ The FRS is basically a sex-specific algorithm which is used to estimate the 10-year CAD risk of an individual.^11^ Estimating risk by FRS is very useful for both individual patient and clinician to decide whether lifestyle modification or preventive medical treatment is required to avoid CAD.

It has been evident in many studies that subjects with impaired glucose tolerance have a substantially increased risk of CAD leading to adverse outcomes.^12^,^13^ Impaired glucose tolerance (IGT) is almost always associated with insulin resistance, a risk factor for CAD.^12^ It has also been observed in different populations that CAD is more prevalent in subjects with IGT as compared to people with normal glycemic controls.^14^ Data for the CAD in IGT subjects is scarcely available for Pakistani Population. Therefore, this study was conducted in order to assess the 10-year risk of coronary artery disease (CAD) in subjects with impaired glucose tolerance (IGT) using Framingham risk score in Karachi, Pakistan.

## METHODS

Data for this study was collected from Diabetes Prevention and Awareness Program. Primary prevention team visited different primary health care centers, factories, service organizations and offices within Karachi, Pakistan. Awareness based educational pamphlets were distributed to the individuals regarding diabetes and primary prevention program.^15^

One thousand eight hundred twenty five people were identified as high-risk and were requested to undertake a standardized oral glucose tolerance test (OGTT). Of these, 1739 subjects agreed to undertake OGTT. It showed that 315 subjects were found to have impaired glucose tolerance (IGT) and were included for the study. IGT was diagnosed according to World Health Organization criteria. After taking informed consent, IGT subjects underwent a detailed anthropometric and medical examination. Information regarding their social-demography, dietary habits and physical activities were also obtained with the help of designed questionnaire. All information was gathered by one-to-one based interview by a trained research representative.^15^ The diagnostic criteria for IGT was based on OGTT. At the time of survey, HbA1c was not used as the diagnostic tool for IGT and Diabetes.^16^

Framingham risk score was used in the study to estimate the 10-year cardiovascular risk of a person. Age, gender, cholesterol, HDL, systolic blood pressure with its treatment status and smoking status are required to calculate FRS. Risk in percentage is estimated on the basis of these scores and then it is categorized into mild, moderate and high risk.^11^ To obtain odds ratio, subjects at moderate or high risk were combined into one category and named “At risk” while subjects with low risk were considered as “Not at risk”. According to WHO criteria for Asians, body mass index (BMI) ≥ 25 kg/m^2^ was classified as obese. Hypertension was defined according to IDF clinical criteria as; blood pressure ≥ 130/85 mmHg or on treatment of previously diagnosed hypertension. Cutoff value for triglyceride high density lipoprotein ratio was taken as <3 for normal and ≥3 for abnormal ratio.^17^,^18^ Ethical approval for the study was obtained from the Institutional Review Board (IRB) of Baqai Institute of Diabetology & Endocrinology (BIDE).

### Statistical analysis

Continuous variables were presented in the form of mean and standard deviation, whereas, categorical variables were presented as frequency with percentage. Independent t-test was used for continuous variables and chi-square test was used for categorical variables. P-value < 0.05 was considered statistically significant. Statistical Package for Social Sciences (SPSS) version 17.0 was used for analyses.

## RESULTS

Baseline characteristics of males (n=208) and females (n=107) are shown in Table-I. Mean age of the participants was 44.1 ± 9.8 years. Mean BMI and waist-hip ratio (WHR) were 27.3 ± 5.0 kg/m^2^ and 0.9 ± 0.5 respectively. Significant differences between males and females were observed in BMI and WHR.

**Table-I:** Baseline characteristics of study population.

*Variables*	*Male*	*Female*	*P-value*	*Overall*
n	208	107	-	315
Age (years)	44.5 ± 10.1	43.2 ± 9.1	0.270	44.1 ± 9.8
Weight (kg)	74.2 ± 13.4	72.0 ± 12.9	0.181	73.5 ± 13.2
Height (cm)	168.9 ± 7.2	155.1 ± 6.4	<0.001	164.2 ± 9.5
Body mass index (kg/m^2^)	26.0 ± 4.3	30.0 ± 5.3	<0.001	27.3 ± 5.0
Waist circumference (cm)	97.3 ± 10.8	91.1 ± 11.8	<0.001	95.2 ± 11.5
Hip circumference (cm)	101.9 ± 10.9	110.4 ± 12.8	<0.001	104.8 ± 12.2
Waist-to-hip ratio (WHR)	0.9 ± 0.1	0.8 ± 0.1	<0.001	0.9 ± 0.1
Systolic BP (mmHg)	121.1 ± 15.2	121.5 ± 17.3	0.832	121.3 ± 15.9
Diastolic BP (mmHg)	84.1 ± 9.9	84.1 ± 11.4	0.949	84.1 ± 10.4

Data presented as Mean ± S.D

Biochemical characteristics of subjects are shown in Table-II. No significant difference was found between males and females except for HDL-cholesterol (p=0.011). Females had significantly higher levels of HbA1c (7.0 ± 0.7) (%) as compared to males (6.3 ± 1.2) with p-value < 0.001.

**Table-II:** Biochemical characteristics of study population.

*Variables*	*Male*	*Female*	*P-value*	*Overall*
n	208	107	-	315
HbA1c (%)	6.3 ± 1.2	7.0 ± 0.7	<0.001	6.5 ± 1.1
Serum Creatinine (mg/dl)	1.3 ± 2.1	0.9 ± 0.2	0.339	1.2 ± 1.7
Fasting blood glucose (mg/dl)	101.5 ± 11.8	102.2 ± 13.6	0.622	101.8 ± 12.4
Random blood glucose (mg/dl)	157.8 ± 17	159.3 ± 15.8	0.451	158.3 ± 16.6
Total cholesterol (mg/dl)	177.9 ± 34.6	180.8 ± 27.8	0.453	178.9 ± 32.5
Triglycerides (mg/dl)	154.8 ± 97.9	152.1 ± 99.8	0.816	153.9 ± 98.4
High density lipoprotein (mg/dl)	37.5 ± 5.3	40.1 ± 12.6	0.011	38.4 ± 8.6
Low density lipoprotein (mg/dl)	115.8 ± 23.4	118.6 ± 21.4	0.299	116.8 ± 22.7
TG/HDL-C	4.4 ± 3.4	4.0 ± 3.2	0.350	4.2 ± 3.3

Data presented as Mean ± S.D Where, TG is triglycerides and HDL-C is high density lipoprotein cholesterol

**Fig.1:**
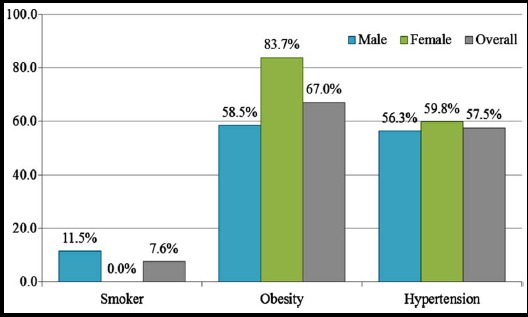
Characteristics of study population. P-value was found significant (p<0.0001) in all cases except for “hypertension” with p-value = 0.545

Characteristics like smoking status, hypertension and obesity is presented in Fig.1. Only 7.6% males were smoker; whereas, percentage of depressed subjects was 20.1%. Majority of the subjects were obese (67.0%) and hypertensive (57.5%). Significant differences between males and females were observed in smoking status and obesity.

**Fig.2:**
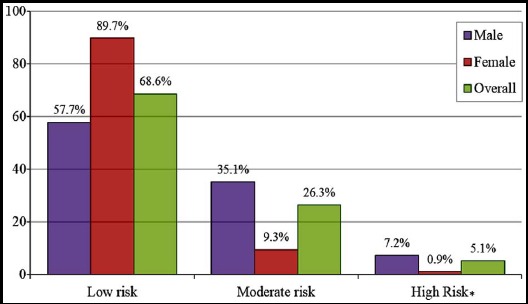
Risk of CAD according to Framingham risk score. P-value was found significant (p<0.05) in all cases.

Fig.2 presents subjects at risk of CAD after 10 years assessed through Framingham risk score assessment. Majority of the IGT subjects (68.6%) were at low risk, followed by 26.3% at moderate and 5.1% at high risk of developing CAD. Percentage of male subjects at moderate and high risk was significantly higher as compared to females (p-value < 0.001).

Table-III presents odds ratio of having CAD against factors including gender, hypertension, obesity, and TG/HDL-C ratio. The results showed that males were 6.4 times more likely to have CAD in next 10 year as compared to females. Similarly being Hypertensive and having TG/HDL-C ≥ 3 carries 2.4 and 2.05 times higher risk to develop CAD.

**Table-III:** Risk of CAD according to Framingham risk score.

*Variables*		*At risk*	*Not at risk*	*P-value*	*Odds ratio*
n (%)		99 (31.4%)	216 (68.6%)	-	-
Gender*	Male	88 (88.9%)	120 (55.6%)	<0.001	6.40 (3.24-12.65)
	Female	11 (11.1%)	96 (44.4%)		
Hypertension*	Yes	71 (71.7%)	110 (50.9%)	0.001	2.44 (1.46-4.08)
	No	28 (28.3%)	106 (49.1%)		
Obesity	Yes	67 (68.4%)	140 (66.4%)	0.726	1.10 (0.66-1.83)
	No	31 (31.6%)	71 (33.6%)		
TG/HDL-C	≥ 3	67 (67.7%)	109 (50.5%)	0.004	2.05 (1.25-3.38)
	< 3	32 (32.3%)	107 (49.5%)		

Data presented as n (%) Where, TG is triglycerides and HDL-C is high density lipoprotein cholesterol Odds ratio (at risk/no risk) was calculated

* denotes significant odds ratio.

## DISCUSSION

According to the findings of this study, it was found that male IGT subjects had 6.4 times more chances of developing CAD than females as shown in previous study done in Karachi in 2008.^3^ Reduced risk observed in females could be because of the comparative better lipid profile along with the unique hormonal protection in reproductive age group and low levels of serum creatinine. Being nonsmoker would further decrease their risk to develop CAD. It is a well-known fact that increased HDL is beneficial for subjects with CAD.^19^,^20^ Around 32% of the subjects with IGT in this study were at risk to develop CAD. Association of diabetes and IGT with deaths from cardiovascular disease has been observed widely.^21^,^22^ Taking in to consideration the rising prevalence of abnormal glucose tolerance, people at risk must be considered a high priority.

Obesity is a major risk factor for developing CAD and diabetes mellitus.^23^,^24^ Subjects in our study were mostly obese (BMI ≥ 25 kg/m^2^),^25^ however, the results of this study suggests that obese and non-obese IGT subjects have equal chances of having CAD suggesting that regardless of the obesity status, all subjects with abnormal glucose tolerance should be focused for their risk of CAD. Hypertensive subjects were 2.4 times more likely to develop CAD than non-hypertensive subjects. It is agreed that raised blood pressure is an independent risk factor both in normal and abnormal glucose tolerant subjects.^26^ Majority of the study subjects especially females, were non-smokers. Clair C et al., found in their study that, in people without diabetes, cessation of smoking lowers the risk of CAD.^19^

## CONCLUSION

In this study, Framingham score helped us to identify that considerable numbers of the subjects with IGT in Karachi Pakistan were at risk to develop CAD. Abnormal glucose tolerance is considered as CAD risk equivalent necessitating the need to diagnose the conditions early in order to decrease morbidity and mortality rate. Further large scale, community based, randomized case-control studies are required to exactly quantify the risk of CAD associated with Pakistani abnormal glucose tolerant population. Proper allocation of resources, policy making and implementation would be the next steps to create awareness in the community at mass levels.
